# Long-term left ventricular assist device support reinforces detrimental immunological effects

**DOI:** 10.3389/fimmu.2026.1705980

**Published:** 2026-02-09

**Authors:** Maja-Theresa Dieterlen, Malina Sauskat, Kristin Klaeske, Eva Katharina Messer, Joanna Jozwiak-Nozdrzykowska, Alexey Dashkevich, Michael A. Borger, Sandra Eifert, Michal Nozdrzykowski

**Affiliations:** 1 University Clinic of Cardiac Surgery, HELIOS Clinic, University of Leipzig, Heart Center Leipzig, Germany; 2 Department of Cardiology, Heart Center Leipzig at Leipzig University, Leipzig, Germany

**Keywords:** dendritic cells, immune system, left ventricular assist device, long-term, NK cell, T-cells

## Abstract

**Introduction:**

Infection is a common complication following left ventricular assist device (LVAD) implantation that increases the mortality in the post-implantation period. Immunological changes affecting dendritic cells (DCs), natural killer (NK) cells and T-cells have been observed in the first year after implantation, but long-term data are missing. We investigated if long-term LVAD support has stronger effects on the immune system than short-term LVAD support.

**Methods:**

Blood samples were obtained from patients with 12–18 months of LVAD support (short-term LVAD; n=53) and from patients with ≥36 months of LVAD support (long-term LVAD; n=57). Flow cytometric analyses for CD4^+^ and CD8^+^ T-cells, T_regs_, B cells, NK cells and DCs were performed. Additionally, terminal differentiation and activation of immune cells was quantified.

**Results:**

Both groups were comparable with regard to sex, age at LVAD implantation, preoperative BMI, etiology of heart failure, NYHA class, left ventricular ejection fraction, INTERMACS, and implant strategy. Platelet counts were lower in the long-term LVAD group (p=0.05). Flow cytometric analyses for CD4^+^ and CD8^+^ T-cells, T_regs_, B-cells, NK cells and DCs revealed that the percentages of total DCs (p<0,01), BDCA3^+^ myeloid DCs (p<0.01), BDCA2^+^ (p=0.01) and BDCA4^+^ plasmacytoid DCs (p=0.02), CD19^+^ B-cells (p<0.01) and of immunoregulatory CD56^bright^ NK cells (p<0.01) were reduced in long-term compared to short-term LVAD patients. Terminal differentiation of NK cells measured by CD57 expression was higher in long-term than in short-term LVAD patients (p=0.03).

**Conclusion:**

This cross-sectional observational study revealed that long-term (≥36 months) LVAD support may contribute to detrimental effects on the immune system in comparison to short-term LVAD support. The long-lasting LVAD support may influence DCs, NK cells and B-cells that show a progression of cellular changes.

## 1 Introduction

The implantation of a left ventricular assist device (LVAD) is a durable therapeutic option for patients with end-stage heart failure. In 2024, 81.4% of the device implantations were indicated as destination therapy (1). The HeartMate 3™ (HM3) device is the most commonly implanted device type with a 5-year survival of 58.4% (2). According to their technical properties, LVADs, including the HM3 device, induce a non-physiological shear stress that could lead to relevant changes of different areas and physiological systems in the human body such as the blood vessels, the coagulation system and the immune cells (3). An ongoing shear stress on circulating immune cells could have an effect on these cells reaching from either an increase or a decrease of the cell proliferations, their activation or maturation (4–7). In addition to natural killer (NK) cells, B- and T-cells, rare immune cell populations such as dendritic cells (DCs), which are antigen-presenting cells and interferon producers that regulate the innate and adaptive immune response, are affected. Changes of circulating immunological cells within the first year after implantation have been described in several studies and comprise the increase of myeloid dendritic cells (mDCs), NK cells, CD8^+^ effector T-cells and the neutrophil-lymphocyte ratio (NLR) as well as a decrease in CD4^+^ T-helper cells and the DC population (4, 5). Further, it has been shown that a number of cytokines such as monocyte chemoattractant protein-1, interferon γ-induced protein, interleukin-8, tumor necrosis factor-α, macrophage-derived chemokine and macrophage inflammatory protein-β increased in the first year after continuous-flow LVAD implantation (8). The incidence of LVAD-specific, LVAD-related and non-LVAD infections is increasing with longer LVAD support (9) which could indicate a progressive degradation of the immunological function.

Observations on immunological changes have not been described for an LVAD support time of more than 12 months. Therefore, it is unclear if long-term LVAD support may contribute to a progression of detrimental immunological effects.

In this study, we compared the immunological changes in patients with long-term (≥ 36 months) LVAD support compared to immunological changes induced after 1 year of LVAD support to investigate if long-term LVAD support influences the immune system compared to short-term LVAD.

## 2 Materials and methods

### 2.1 Study population

The present study was approved by the Ethics Committee of the Medical Faculty, University of Leipzig, Germany (vote numbers: ID:225/17-ek and 321/23-ek), and was performed according to the guidelines of the Declaration of Helsinki (2013). The patients were consecutively recruited in the LVAD outpatient clinic at the Department for Cardiac Surgery at the Heart Center Leipzig after checking the inclusion and exclusion criteria. Inclusion criteria were: age ≥ 18 years, HM3 implantation and written informed consent. Exclusion criteria were: refusal of written informed consent, pregnancy, known autoimmune (e.g. Hashimoto thyroiditis, Lupus erythematodes, rheumatoid arthritis etc.), immunological (e.g. human immunodeficiency virus infection etc.) or malignant diseases (e.g. breast cancer, lymphoma etc.), and active infection with clinical symptoms. Patients gave their written informed consent. Following blood withdrawal, blood samples were pseudonymized to ensure a blinded analysis. This cross-sectional observational study included 110 patients who received an LVAD implantation of the HeartMate 3™ (HM3) device (Abbott, Chicago, IL, USA) at the Heart Center Leipzig, Germany. The short-term LVAD group consisted of n = 53 patients with a LVAD support of 12–18 months, the long-term LVAD group consisted of n = 57 patients with an LVAD support of at least 36 months. There was no overlap of patients between the short-term and the long-term group.

### 2.2 Demographic and clinical characteristics

Demographic and clinical data were collected. The sex, preoperative body mass index (BMI), etiology of heart failure, NYHA classification, preoperative left ventricular ejection fraction (LVEF), INTERMACS score and the indication of LVAD implantation were documented. Further, comorbidities and blood count parameters, the International Normalized Ratio (INR), and the NLR were analyzed at the time point of LVAD implantation.

### 2.3 Blood sampling and blood count analysis

Citrated blood samples were collected and used for flow cytometric analyses of immunological cell populations. Blood count analysis was conducted by the MVZ Laboratory Dr. Reising-Ackermann (Leipzig, Germany) and included the measurement of the hematocrit, hemoglobin, erythrocytes, leukocytes, platelets, lymphocytes, monocytes, neutrophils, C-reactive protein (CRP), and INR.

### 2.4 Flow cytometry

Citrated blood samples were treated as described previously (10, 11). Total CD19^+^ B-cells, CD3^+^ T-cells, CD4^+^ T-helper cells and CD8^+^ effector T-cells as well as the degree of terminal differentiation/senescence (CD57) and activation (CD25) were quantified. T_regs_ were defined as CD3^+^CD4^+^ cells with a high CD25 expression and a low CD127 expression. Peripheral blood dendritic cell (DC) subsets were defined as lineage cocktail-1^-^ and HLA-DR^+^ cells. The expression of different blood dendritic cell antigens (BDCAs) was performed to analyze BDCA1^+^ and BDCA3^+^ myeloid DCs (mDCs) and BDCA2^+^ and BDCA4^+^ plasmacytoid DCs (pDCs). Gating strategies were reported by Klaeske et al. (4) and shown in **Supplementary Figures 1-4**.

Staining of immunological cell subsets was performed with antibodies purchased from BD Biosciences (Franklin Lakes, NJ, USA) and BioLegend (San Diego, CA, USA). 200-300 µL of citrated blood was incubated with different antibody panels for 20 min at room temperature: panel A: lineage cocktail-1, HLA-DR, BDCA4, BDCA2; panel B: lineage cocktail-1, HLA-DR, BDCA3, BDCA1; panel C: CD3, CD4, CD25, CD8, CD57; panel D: CD3, CD4, CD25, CD127; panel E: CD3, CD19, CD16, CD56, CD57. Cells were lysed with 2 mL of FACS lysing solution (BD Biosciences) for 10 min. After centrifugation at 300 *g for 5 min at room temperature, the cells were washed with 4 mL phosphate-buffered saline (PBS). The cells were fixed by adding 500 µL of 1% formalin-PBS. Analysis was performed using a *BD FACSCelesta™ Cytometer* and *BD FACSDiva version 9.2* software *(*both BD Biosciences). Instrument standardization was performed by weekly measurements of Cytometer Setup and Tracking Beads (BD Biosciences).

### 2.5 Statistics

Data were collected and analyzed using Microsoft Excel 2016 software (Microsoft Corporation, Redmond, WA, USA) and SPSS Statistics 28 software (IBM Corp., New York, USA, 1989). Missing data were not replaced; an imputation technique was not applied. Unless stated otherwise, data are presented as mean ± standard deviation for continuous data or as percentage proportion for categorical variables. The comparison of means of the study groups was performed using the T test for metric parameters and the Pearson χ² test or the Yates continuity correction in case of categorical data. Levene’s test was used to test the homogeneity of variance. For all analyses, p-values ≤ 0.05 were considered as statistically significant. The calculation of the number of cases for this study based on a power of 0.8 and an assumed effect size of 1.1, that was calculated from a preliminary study. A minimum of n = 14 patients per group was required.

## 3 Results

### 3.1 Clinical characteristics

The patients in both groups were predominantly male (**Table 1**). LVAD support duration was 1.0 ± 0.2 yrs in the short-term group and 5.5 ± 1.7 yrs in the long-term group. LVAD parameters such as speed (p = 0.71), power (p = 0.81), pump flow (p = 0.42) and pulsatile index (p = 0.20) at the time point of immune status measurement were comparable between both groups (**Supplementary Table 1**). Both groups were comparable with regard to sex, age at LVAD implantation, preoperative BMI, etiology of heart failure, NYHA class, LVEF, INTERMACS, and implant strategy (**Table 1**). The comorbidities arterial hypertension, hyperlipoproteinemia, diabetes mellitus type 2, chronic kidney disease, hypothyroidism, chronic inflammatory disease and chronic obstructive pulmonary disease/bronchial asthma at the time of LVAD implantation were comparable between the groups (**Supplementary Table 2**). In addition, the history of valve surgery, cerebrovascular accidents, malignant diseases, chemotherapy or radiation, drug abuse, intolerances and alcohol and nicotine consumption did not differ between the groups (**Supplementary Table 2**). Detailed information about LVAD-specific, LVAD-related and non-LVAD infections after LVAD implantation in the short-term and the long-term group were reported in **Table 2**. Furthermore, we documented N-terminal pro-B-type natriuretic peptide (NT-proBNP) levels and the right ventricular function at the time point of immune status analysis in the short-term and the long-term group and found comparable levels of NT-proBNP (short-term LVAD: 3,573 ng/L [95% CI: 1,005; 6,142], long-term LVAD: 2,966 ng/L [95% CI: 1,979; 3,954], p = 0.65) and comparable rate of right heart failure (short-term LVAD: 41.5%, long-term LVAD: 36.8%, p = 0.76). According to the lactate dehydrogenase (LDH; short-term LVAD: 4.05 µL/L*s [95% CI: 3.83; 4.27], long-term LVAD: 4.03 µL/L*s [95% CI: 3.77; 4.28], p = 0.91) and bilirubin levels (short-term LVAD: 10.7 µM/L [95% CI: 9.2; 11.3], long-term LVAD: 11.3 µL/L*s [95% CI: 9.6; 13.0], p = 0.61), the intensity of shear stress is comparable between short-term and long-term LVAD group.

**Table 1:** Demographic and clinical characteristics prior to LVAD implantation in the short-term and long-term LVAD group.

Parameter	Short-term LVAD n = 53	Long-term LVAD n = 57	p value
male gender	44 (83.0%)	45 (78.9%)	0.76
age at LVAD implantation [yrs]	59.7 ± 9.3 (95% CI: 57.1, 62.2)	61.9 ± 9.7 (95% CI: 59.3, 64.5)	0.23
preoperative BMI [kg/m^2^]	29.5 ± 5.5 (95% CI: 28.0, 31.1)	27.8 ± 5.0 (95% CI: 26.4, 29.2)	0.09
etiology			1
IHD	31 (58.5%)	33 (57.9%)	
DCM	22 (41.5%)	24 (42.1%)	
NYHA classification			0.12
NYHA III	28 (52.8%)	19 (35.8%)	
NYHA IV	25 (47.2%)	34 (64.2%)	
preoperative LVEF [%]	20.4 ± 6.0 (95% CI: 18.8, 22.1)	18.9 ± 5.8 (95% CI:17.3, 20.5)	0.18
INTERMACS*			0.85
1	6 (11.3%)	7 (13.2%)	
2	9 (17.0%)	12 (22.6%)	
3	20 (37.7%)	22 (41.5%)	
4	12 (22.6%)	9 (17.0%)	
5	2 (3.8%)	1 (1.9%)	
6	4 (7.5%)	2 (3.8%)	
indication for LVAD implantation			0.43
bridge to transplant	7 (13.2%)	4 (7.0%)	
bridge to decision	25 (47.2%)	25 (43.9%)	
destination therapy	21 (39.6%)	28 (49.1%)	

*missing data for n = 4 in the long-term group. The comparison of means was performed using the T test for metric parameters and the Pearson χ² test or the Yates continuity correction in case of categorical data. Levene’s test was used to test the homogeneity of variance. BMI, body mass index; CI, confidence interval; DCM, dilative cardiomyopathy; HTx, heart transplantation; IHD, ischemic heart disease; INTERMACS, Interagency Registry for Mechanically Assisted Circulatory Support; CI, confidence interval; LVAD, left ventricular assist device; LVEF, left ventricular ejection fraction; NYHA, New York Heart Association.

**Table 2:** History of infection after LVAD implantation in the short-term and long-term LVAD groups.

Type of infection	Short-term LVAD n = 53	Long-term LVAD n = 57
LVAD-specific infections		
percutaneous DL infection	20 (37.7%)	28 (49.1%)
pocket infection	0 (0%)	0 (0%)
pump/canula infection	0 (0%)	0 (0%)
LVAD-related infections		
infectious endocarditis	2 (3.8%)	3 (5.3%)
mediastinitis	0 (0%)	0 (0%)
ICD pocket infection	0 (0%)	1 (1.8%)
paracardial abscess	0 (0%)	1 (1.8%)
pathogens detected in BC	7 (13.2%)	6 (10.5%)
non-LVAD infections		
pulmonary infection	9 (17.0%)	31 (54.4%)
*Clostridium difficile* infection	1 (1.9%)	3 (5.3%)
urinary infection	9 (17.0%)	22 (38.6%)
other infection	7 (13.2%)	20 (35.1%)
pathogens detected in BC	7 (13.2%)	16 (28.1%)

Categorical parameters were analyzed using the Pearson χ² test or the Yates continuity correction. BC, blood culture; CMV, cytomegalovirus; DL, driveline; ICD, implantable cardioverter-defibrillator; LVAD, left ventricular assist device.

### 3.2 Blood count analysis

Blood analyses in the short-term and long-term LVAD group at the time point of immunological evaluation revealed comparable values of hemoglobin (p = 0.70), hematocrit (p = 0.19), the cellular blood components except for platelets, the neutrophil-lymphocyte ratio (p = 0.41) and INR values (p = 0.41) (**Table 3**). Platelet counts were within the reference range in both groups, but were lower in the long-term LVAD group (p = 0.05) (**Table 3**). CRP values were comparable between short-term (7.4 ± 14.0 mg/L [95% CI: 3.4, 11.3 mg/L] and long-term LVAD patients (7.1 ± 10.0 mg/L [95% CI: 4.4, 9.7 mg/L], p = 0,88).

**Table 3:** Blood count parameters in the short-term and long-term LVAD group.

Blood count parameter	Reference values	Short-term LVAD n = 53	Long-term LVAD n = 57	p value
hemoglobin [mmol/L]	♀ 7.6-9.5 ♂ 8.7-10.9	8.3 ± 1.3 (95% CI: 7.9, 8.6)	8.4 ± 1.3 (95% CI: 8.0, 8.7)	0.70
hematocrit	♀ 0.35-0.47 ♂ 0.40-0.52	0.40 ± 0.61 (95% CI: 0.38, 0.41)	0.41 ± 0.62 (95% CI: 0.39, 0.43)	0.19
erythrocytes [Tpt/L]	♀ 4.1-5.1 ♂ 4.5-5.9	4.4 ± 0.7 (95% CI: 4.3, 4.6)	4.4 ± 0.7 (95% CI: 4.2, 4.6)	0.67
platelets [Gpt/L]	150-400	215 ± 71 (95% CI: 195, 234)	182 ± 49 (95% CI: 169, 195)	0.05
INR	2.0-2.5	2.18 ± 0.47 (95% CI: 2.05, 2.32)	2.25 ± 0.33 (95% CI: 2.16, 2.34)	0.41
leukocytes [Gpt/L]	4.4-11.3	8.1 ± 2.5 (95% CI: 7.5, 8.8)	7.5 ± 2.0 (95% CI: 7.0, 8.0)	0.14
neutrophils [Gpt/L]	2.2-6.3	5.9 ± 2.1 (95% CI: 5.3, 6.5)	5.4 ± 1.7 (95% CI: 4.9, 5.9)	0.16
neutrophils [%]*	50-70	71 ± 7 (95% CI: 69, 73)	71 ± 8 (95% CI: 69, 73)	0.97
lymphocytes [Gpt/L]	1.00-4.10	1.42 ± 0.52 (95% CI: 1.27, 1.57)	1.26 ± 0.52 (95% CI: 1.13, 1.40)	0.12
lymphocytes [%]*	25-49	18 ± 6 (95% CI: 16, 19)	17 ± 7 (95% CI: 16, 19)	0.77
monocytes [%]*	0-10	9 ± 2 (95% CI: 8, 10)	10 ± 2 (95% CI: 9, 10)	0.23
NLR	0.8-3.5	4.6 ± 2.3 (95% CI: 4.0, 5.3)	5.1 ± 3.2 (95% CI: 4.2, 5.9)	0.41

The comparison of means was performed using the T test for metric parameters. Levene’s test was used to test the homogeneity of variance. ♀, female sex; ♂, male sex; CI, confidence interval; LVAD, left ventricular assist device; INR, international normalized ratio; NLR, neutrophil-lymphocyte ratio; * percentage of leukocytes.

### 3.3 Flow cytometric analysis of innate immunity parameters

**Figure 1:**
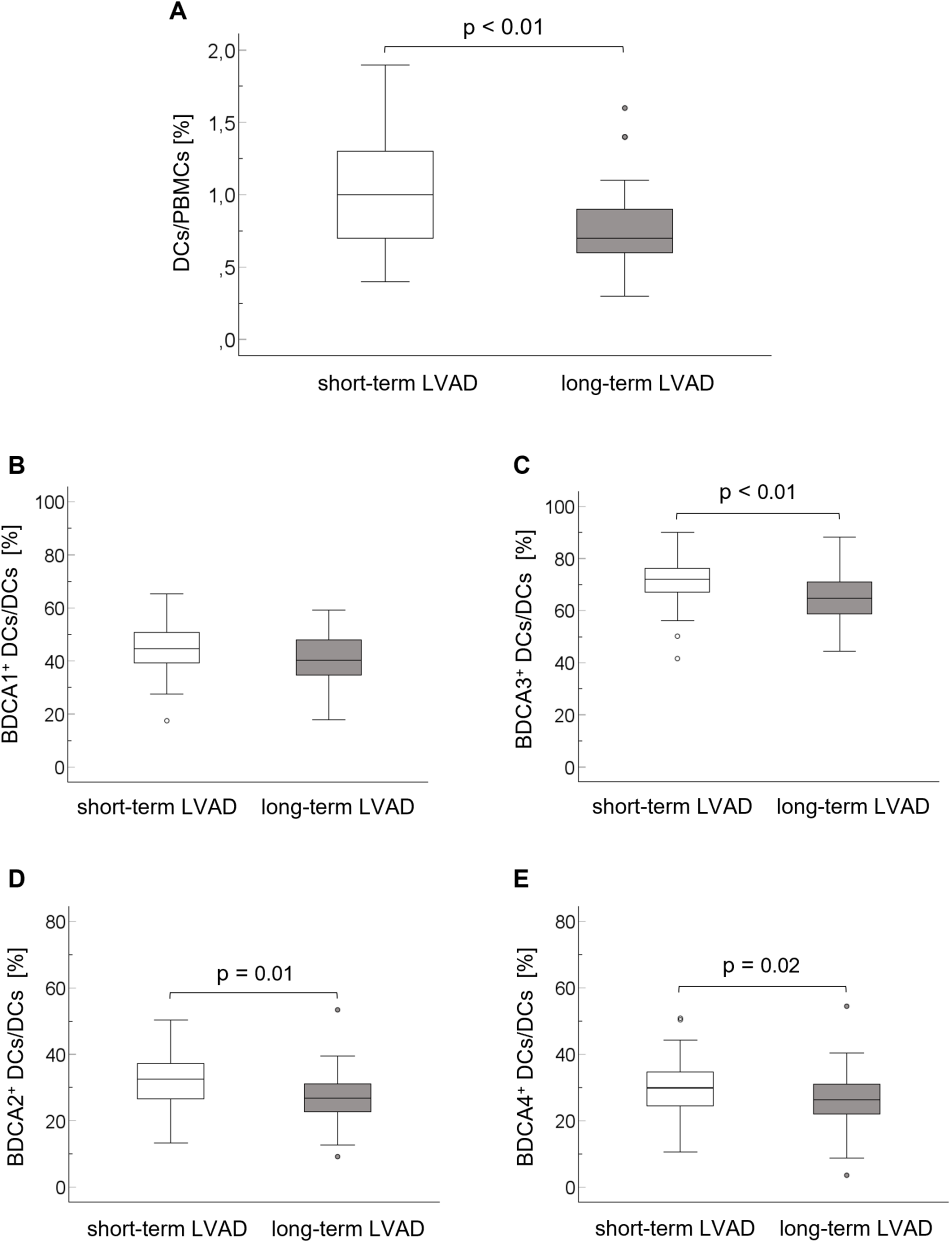
Proportions of DCs on PBMCs **(A)** and BDCA1^+^ DCs **(B)**, BDCA2^+^ DCs **(C)**, BDCA3^+^ DCs **(D)** und BDCA4^+^ DCs **(E)** on total DCs in the short-term and long-term LVAD group. The comparison of means was performed using the T test for metric parameters. Levene’s test was used to test the homogeneity of variance. BDCA, blood dendritic cell antigen; DCs, dendritic cells; IQR, interquartile range; LVAD, left ventricular assist device; PBMCs, peripheral blood mononuclear cells.

The flow cytometric analysis of the innate immune system comprised DC and NK subsets. In our study, the population of total DCs was lower in long-term LVAD patients (0.73 ± 0.27% [95% CI: 0.65, 0.80] than in short-term LVAD patients (1.01 ± 0.40% [95% CI: 0.90], p < 0.01) (**Figure 1A**). The analyses of BDCA1^+^ and BDCA3^+^ mDCs revealed that the proportion of BDCA1^+^ mDCs was comparable between the groups (short-term LVAD: 45.0 ± 9.8% [95% CI: 42.3, 47.7], long-term LVAD: 41.6 ± 9.5% [95% CI: 39.1, 44.1], p = 0,07), but the proportion of myeloid BDCA3^+^ DCs was lower in the long-term (64.5 ± 9.5% [95% CI: 61.9, 67.0]) compared to short-term LVAD group (71.4 ± 8.9% [95% CI: 68.9, 73.8], p < 0.01) (**Figures 1B, C**). The proportions of BDCA2^+^ and BDCA4^+^ pDCs were lower in long-term LVAD patients (BDCA2^+^: 27.3 ± 7.6% [95% CI: 25.3, 29.3]; BDCA4^+^: 26.4 ± 8.5% [95% CI: 24.1, 28.6]) compared to short-term LVAD patients (BDCA2^+^: 31.3 ± 8.4% [95% CI: 29.0, 33.6], p = 0.01; BDCA4^+^: 30.2 ± 8.7% [95% CI: 27.8, 32.6], p = 0.02) (**Figures 1D, E**).

**Figure 2:**
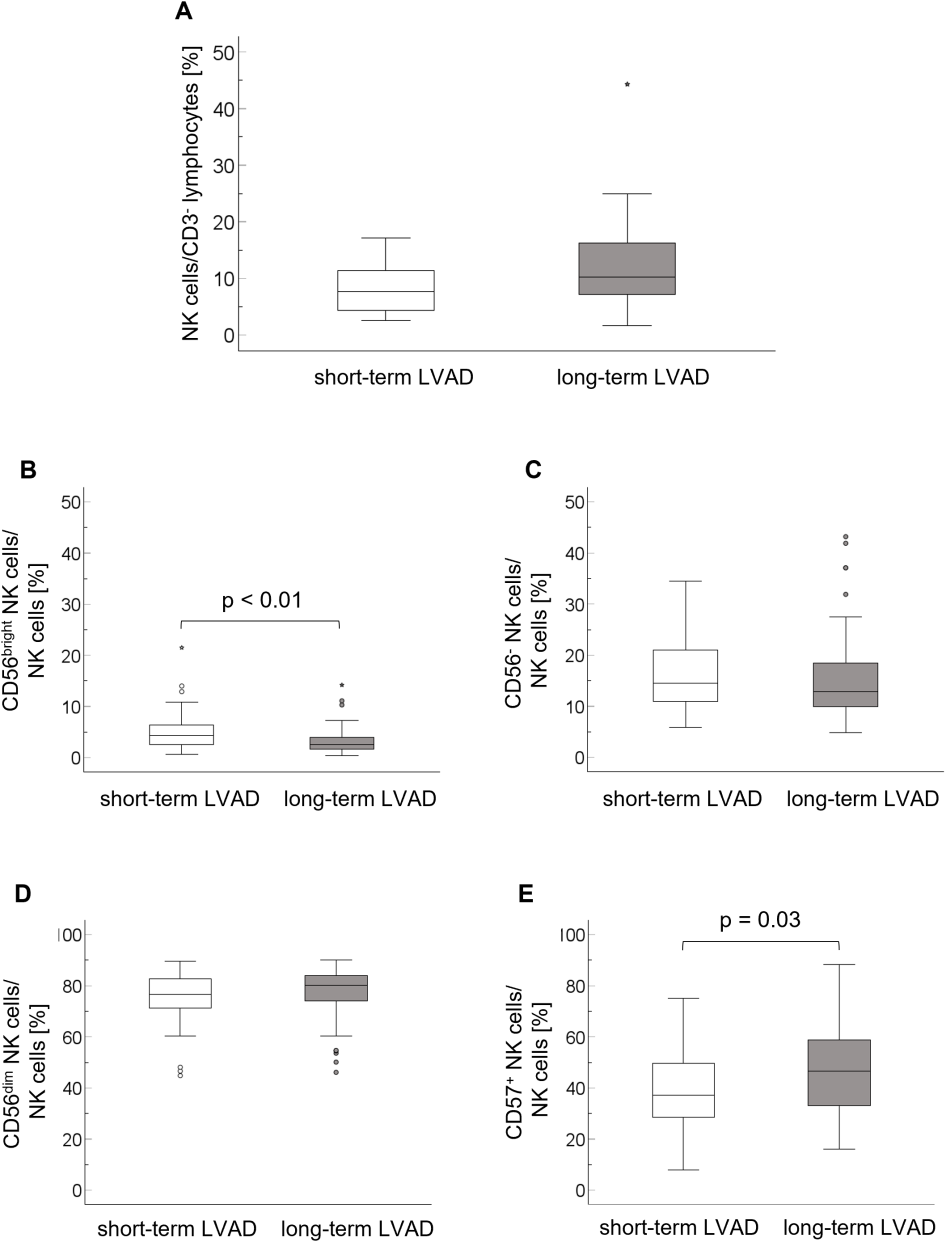
Proportions of NK cells on CD3^-^ lymphocytes **(A)** and the CD56^bright^**(B)**, CD56^-^**(C)**, CD56^dim^**(D)** NK cell subpopulations and the CD57 expression **(E)** on total NK cells in the short-term and long-term LVAD group. The comparison of means was performed using the T test for metric parameters. Levene’s test was used to test the homogeneity of variance. IQR, interquartile range; LVAD, left ventricular assist device; NK cells, natural killer cells.

The population of NK cells was comparable between the groups (short-term LVAD: 8.5 ± 4.9% [95% CI: 5.0, 12.0], long-term LVAD: 12.3 ± 7.5% [95% CI: 10.3, 14.4], p = 0.13) (**Figure 2A**). The proportion of immunoregulatory CD56^bright^ NK cells was lower in long-term LVAD patients (3.3 ± 2.6% [95% CI: 2.6, 4.0]) compared to short-term LVAD patients (5.2 ± 3.7% [95% CI: 4.1, 6.2], p < 0.01) (**Figure 2B**). CD56^-^ NK cells (short-term LVAD: 16.2 ± 7.3% [95% CI: 14.2, 18.2], long-term LVAD: 15.5 ± 8.9% [95% CI: 13.1, 17.9], p = 0.66) and CD56^dim^ NK cells (short-term LVAD: 75.1 ± 10.4% [95% CI: 72.2, 78.0], long-term LVAD: 77.3 ± 10.5% [95% CI: 74.5, 80.1], p = 0.27) were comparable between the groups (**Figures 2C, D**). Terminal differentiation of NK cells measured by CD57 expression was higher in long-term (46.4 ± 18.4% [95% CI: 41.5, 51.3]) than in short-term LVAD patients (39.1 ± 16.5% [95% CI: 34.6, 43.7], p = 0.03) (**Figure 2E**).

### 3.4 Flow cytometric analysis of adaptive immunity parameters

The flow cytometric analysis of the adaptive immune system comprised CD3^+^ T-cells, CD4^+^ and CD8^+^ T-cells, T_regs_ and CD19^+^ B-cells. The proportion of CD3^+^ T-cells was comparable between the groups (short-term LVAD: 24.6 ± 5.9% [95% CI: 20.4, 28.9], long-term LVAD: 30.4 ± 11.7% [95% CI: 27.3, 33.5], p = 0.07). The proportions of the T-cell subsets CD8^+^ effector T-cells (p = 0.97), CD4^+^ T-helper cells (p = 0.80) and CD4^+^ CD25^high^ CD127^low^ T_regs_ (p = 0.50) were comparable between short-term and long-term LVAD patients (**Table 4**). Terminal differentiation measured by CD57 expression and T-cell activation measured by CD25 expression in CD8^+^ effector T-cells and CD4^+^ T-helper cells were comparable between the groups (**Table 4**).

**Table 4:** Percentages of T-cell subpopulations, the senescence (CD57) and activation status (CD25) in the short-term and long-term LVAD group. Percentages are related to the CD3^+^ T-cell population.

T-cell population	Short-term LVAD n = 53	Long-term LVAD n = 57	p value
CD8^+^ T-cells [%]	31.1 ± 11.6 (95% CI: 27.9, 34.2)	31.0 ± 14.9 (95% CI: 27.0, 34.9)	0.97
CD8^+^/CD57^+^ T-cells [%]	32.9 ± 16.4 (95% CI: 28.4, 37.5)	36.0 ± 18.1 (95% CI: 30.9, 41.0)	0.19
CD8^+^/CD25^+^ T-cells [%]	23.9 ± 8.8 (95% CI: 17.6, 30.2)	19.1 ± 12.4 (95% CI: 15.6, 22.6)	0.13
CD4^+^ T-cells [%]	63.7 ± 12.4 (95% CI: 60.3, 67.1)	63.0 ± 16.2 (95% CI: 58.7, 67.3)	0.80
CD4^+^/CD57^+^ T-cells [%]	7.6 ± 8.4 (95% CI: 5.3, 9.9)	6.3 ± 8.7 (95% CI: 4.0, 8.6)	0.44
CD4^+^/CD25^+^ T-cells [%]	70.2 ± 15.4 (95% CI: 59.2, 81.2)	74.6 ± 12.7 (95% CI: 70.9, 78.2)	0.34
T_regs_ [%]	8.5 ± 2.5 (95% CI: 7.8, 9.2)	8.2 ± 2.6 (95% CI: 7.5, 8.9)	0.50

The comparison of means was performed using the T test for metric parameters. Levene’s test was used to test the homogeneity of variance. CD, cluster of differentiation; CI, confidence interval; LVAD, left ventricular assist device; T_regs_, regulatory T-cells.

In addition to T-cells, B-cells were quantified by flow cytometry. The proportion of CD19^+^ B-cells was lower in long-term (6.2 ± 4.5% [95% CI: 4.9, 7.4]) than in short-term LVAD patients (14.6 ± 9.5% [95% CI: 12.0, 17.2], p < 0.01).

## 4 Discussion

This study compared the immunological changes in patients with long-term (≥ 36 months) LVAD support with those induced after one year of LVAD support, to investigate whether long-term LVAD support influences the immune system compared to short-term LVAD support. We found immunological changes in the innate and the adaptive immune system comprising lower percentages of total DCs, BDCA3^+^ mDCs, BDCA2^+^ and BDCA4^+^ pDCs, CD56^bright^ NK cells and B-cells as well as an increase of the CD57 expression in the NK cell population in long-term LVAD patients compared to short-term LVAD patients. The results of the present study should be interpreted as hypothesis-generating, rather than as evidence of progressive immune deterioration.

Blood count analyses provide an overview of blood cell types and their characteristics, but are not suitable to detect differences for low-percentage leukocyte subpopulations of the immune system such as NK cells and DCs. Thus, in LVAD patients, blood count analyses do not uncover immunological changes of leukocytes that could be relevant for an increased infection risk in this patient population.

In our study, platelets were lower but within the reference range in patients with long-term LVAD group compared to short-term LVAD. Platelet reduction and thrombocytopenia are common following LVAD implantation, affecting up to 36% of women and up to 24% of men with an implanted LVAD (12). The majority of the studies investigated the effects of platelet reduction following LVAD implantation regarding bleeding complications. However, platelets have an essential role in the immune system, interact with antigen-presenting cells, T-cells, NK cells, and influence the cytokine and chemokine secretion during inflammation and pathogen inactivation (13–15). Therefore, it is possible that the lower platelet count in LVAD patients is related to immunological changes in this patient cohort.

Furthermore, experimental and clinical evidence is indicating that platelets promote tumor progression and metastasis through a wide range of physical and functional interactions between platelets and cancer cells (16).

Our study demonstrated that long-term LVAD support may lead to a decrease of the total DC population. This decrease affects either BDCA3^+^ mDCs or BDCA2^+^ and BDCA4^+^ pDCs. The detailed analysis of DC subsets using BDCA surface markers is necessary to distinguish between the subtype-specific different functions and origins of this cell population.

DCs are referred to as “guardian cells” of the immune system, because they are able to bind and process antigens and pathogens, and present them to T-cells (17). Further, DCs produce cytokines and interferons that regulate T-cells and maintain the immunological tolerance (17, 18). While BDCA3^+^ mDCs can activate CD8^+^ T cells, BDCA1^+^ mDCs display the conventional DC subset involved in regulating the humoral immune response (17–21). BDCA2^+^ and BDCA4^+^ pDCs are producers of interferons and promote CD4^+^ T-cell activation, migration and survival (17–20). A reduction of DCs could result in a reduced antigen presentation and ability to activate CD8^+^ and CD4^+^ T-cells. This, in turn, could impede pathogen elimination and increase the infection risk and duration.

We could not detect a reduced level of activation in CD4^+^ and CD8^+^ T-cells according to the measurements of CD25 expression of long-term LVAD patients in our study. Nevertheless, the observed cell depletion of total DCs and of three out of four investigated DC subsets in long-term LVAD patients suggests that long-term LVAD support could lead to further impairment of the immunological defense. This assumption is supported by animal and human data documenting an association between systemic DC depletion and an impairment of patient’s defense mechanisms (22, 23).

Previous investigations reported that DCs were reduced in patients prior to LVAD-implantation compared to healthy, age-matched individuals followed by an increase of mDCs in the first year after LVAD-implantation (24). Our data indicate that the long-term LVAD-support may reverse the first immune-stabilizing effects of the LVAD. Furthermore, future studies should clarify the impact of right heart failure, which affects 39% of the LVAD patients in our study at the time point of immune status analysis, on the immune system and their cell subsets.

Our present study showed that, in addition to DC reduction, NK cells underwent a reduction of the CD56^bright^ NK cell subset as well as an increase of the CD57 expression in long-term compared to short-term LVAD support. CD56^bright^ NK cells, as well as CD56^dim^ NK cells, execute cytotoxicity with a rapid release of cytokines, perforins and granzymes (25). Additionally, CD56^bright^ NK cells have an immunosuppressive and -regulating role which is transmitted via cytokines such as interferon γ, TNF-β, IL-10 and IL-13 (25). An increased expression of CD57 in NK cells indicates an increase in maturity and cytotoxic potential (26). CD57 is expressed by CD16^+^/CD56^dim^ and CD16^+^/CD56^-^ NK cells, but not in CD56^bright^ cells (26). Thus, the analysis of CD57 expression allows conclusions on the cytotoxic potential of CD56^dim^ and CD56^-^ NK cells but not for the CD56^bright^ NK cell subset. Our NK cell analyses indicate that long-term LVAD support may result in an increase of NK cell maturity and a decrease of CD56^bright^ NK cell-mediated cytotoxicity and immunomodulation which could be a cellular response to the changes in the DC subsets. It is known that DC and NK cell subsets depend on each other, because DCs can activate NK cells, and NK cells provide signals for DC activation, maturation and cytokine production (27). A reduction of DC subsets could lead to a reduction of NK cell activation as it has been documented according to the CD57 expression analysis; and a reduction of NK cells could reduce DC maturation which could lead to decreased percentages of mDC and pDC populations. This hypothesis of an impaired DC/NK cell interaction in LVAD patients is speculative and should be proven by functional assays.

DCs and NK cells have major roles in the antiviral defense (28, 29). In LVAD patients, a high percentage of the infections occurring after implantation are bacterial (30). Thus, there might be further immunological changes that we did not detect so far, or DCs and NK cell changes have a stronger effect on bacterial defense mechanisms as known so far.

In our study, CD19^+^ B-cells were reduced in patients with long-term LVAD compared to short-term LVAD. Circulating CD19^+^ B-cells consist of several B-cell subsets including transitional, naïve and memory B-cells (31). Reduced concentrations of circulating B-cells are associated with worse outcomes and an increased risk of infections (31, 32). Reduced CD19^+^ B-cells prior to LVAD implantation have been found in patients with infection in the early post-operative period following LVAD implantation (11) which supports the association with worse outcomes and an increased infection risk in this patient cohort.

The present study did not include the analyses of B cell exhaustion markers under LVAD support. A wide range of methods are available to study this purpose reaching from gene-pair signatures to the surface expression of inhibitory molecules such as PD-1 and FcRL4 (33, 34).

It is known that an LVAD implantation and the resulting contact to foreign material of the LVAD device leads to a hyperreactivity of B-cells that is connected to an increased production of antibodies against auto-antigens and anti-human leukocyte antigens (HLA) (32, 35). In long-term patients, the permanent shear stress and contact to the LVAD device could lead to excessive activation followed by exhaustion of the B-cells and an increased elimination of these cells.

The incidence of infection following LVAD implantation ranges from an increase with longer LVAD support (8) to a decrease (36). When correlating the immunological changes with the infection incidence in LVAD patients it has to be taken into account that LVAD patients and their caregivers increase their expert knowledge and experience with the LVAD device with longer support duration. This applies not only to the technical care (e.g. changing the driveline dressing) and medication management (e.g. antiplatelet therapy and anticoagulation) but also to the daily hygiene to avoid an increase for infection. This learning effect has to be taken into account when interpreting the infection incidence in LVAD patients.

## 5 Limitations

Immune cells have been characterized by their phenotypes defined by the expression of surface molecules. Functional assays have not been performed and should be part of future studies to investigate if, in addition to the reduction of the percentages, the functionality of immune cell populations is reduced. The investigation of the immune cell functionality fully depicts the complexity of immune dysfunction and supplements conventional immunmonitoring (37).

Furthermore, in our study, detailed analyses of monocytes and macrophages have not been performed. Especially bloodstream monocytes would be interest, because they mediate the antimicrobial defense and can differentiate into tissue macrophages and DCs (38).

Investigations on these cell populations would increase the knowledge of LVAD-induced effects on the innate immune system. While the comparison of a short-term and a long-term LVAD cohort in this cross-sectional study allows to draw conclusions about immunological parameter associations, a longitudinal study design would allow to detect causal relationships between the immunological parameters. In addition, baseline measurements at the time point of LVAD implantation are missing. Therefore, it can’t be excluded the baseline immune status is comparable. We reported comorbidities, demographic and clinical characteristics, which were comparable between the study groups and did not indicate differences within the study cohort.

Both groups of this study included a small percentage of patients with chronic inflammatory diseases that do not have an autoimmune/immunological/malignant background. These chronic inflammatory diseases included diseases of the thoracic and abdominal organs (e.g. bronchitis, colitis) and the skin (e.g. atopic dermatitis). We did not exclude these patients, however, their inclusion could bias the interpretation of the immunological analyses.

The multiple immunological parameters analyzed in this study were not adjusted for multiple comparisons, because immunological parameters often show correlations. Bonferroni correction is not indicated when dealing with correlated test statistics and could lead to high type II error rates. The readers are encouraged to weigh the conclusions of the present study.

Furthermore, to improve the clinical application of a patient-specific immune status evaluation, an immune score should be developed allowing the assessment of immunological measurement on one single value. This immune score could be used for multivariate regression analyses to identify independent variables related to the immune status. One possibility for an immune score was reported by Sparks et al. (39). In awareness that differences between individuals in immune parameters tend to dominate over those attributable to disease conditions, they developed a machine learning-based score that tracked disease activities and treatment responses and marked aging in individuals. The score is provided on a web platform for calculations for external datasets to empower precision medicine (39).

## 6 Conclusions

This study revealed that long-term (≥ 36 months) LVAD support may contribute to detrimental effects on the immune system compared to short-term LVAD support. The long-lasting LVAD support may influence DCs, NK cells and B-cells that show a progression of cellular changes. These results improve the knowledge about the immunological changes during LVAD support, and clinicians could prove if the duration of LVAD support should be shortened. For patients without the opportunity for LVAD weaning and high infection rates or recurrent infections, immunomodulatory strategies should be developed to decrease the risk of infection and the risks and costs for infection therapies.

## Data Availability

The data that support the findings of this study are available from the corresponding author upon reasonable request. The data are not publicly available due to ethical restrictions.
