# Supplementary material for: Long-term left ventricular assist device support reinforces detrimental immunological effects
Source: Front Immunol. 2026 Feb 9;17:1705980. doi: 10.3389/fimmu.2026.1705980 (PMC12926156; doi:10.3389/fimmu.2026.1705980)
Supplement: Supplementary file 1 [file DataSheet1.docx]

Supplementary Material

# Supplementary Tables

**Supplementary Table 1: LVAD parameters at the time point of immune status measurement in the short-term and long-term LVAD group.**

|  | short-term LVAD  n = 53 | long-term LVAD  n = 57 | p value |
| --- | --- | --- | --- |
| speed (rpm) | 5234 ± 281  (95% CI: 5157, 5311) | 5213 ± 301  (95% CI: 5133, 5293) | 0.71 |
| power (W) | 3.82 ± 0.44  (95% CI: 3.70, 3.95) | 3.84 ± 0.47  (95% CI: 3.72, 3.97) | 0.81 |
| pump flow (L/min) | 4.96 ± 4.95  (95% CI: 3.60, 6.33) | 4.43 ± 0.58  (95% CI: 4.27, 4.58) | 0.42 |
| pulsatile index | 4.5 ± 1.7  (95% CI: 4.0, 4.9) | 4.1 ± 1.4  (95% CI: 3.7, 4.4) | 0.20 |

Footnote Supplementary Table 1: Metric parameters were analysed using the T test. LVAD, left ventricular assist device; rpm, rounds per minute

**Supplementary Table 2: Comorbidities prior to LVAD implantation in the short-term and long-term LVAD group.**

|  | short-term LVAD  n = 53 | long-term LVAD  n = 57 | p value |
| --- | --- | --- | --- |
| arterial hypertension | 48 (90.6%) | 44 (77.2%) | 0.10 |
| hyperlipoproteinemia | 35 (66.0%) | 38 (66.7%) | 1 |
| diabetes mellitus type 2 | 25 (47.2%) | 24 (42.1%) | 0.73 |
| chronic kidney disease  grade I  grade II  grade III  grade IV | 1 (1.9%)  16 (30.2%)  16 (30.2%)  2 (3.8%) | 1 (1.8%)  16 (28.1%)  17 (29.8%)  2 (3.5%) | 0.91 |
| hypothyroidism | 8 (15.1%) | 10 (17.5%) | 0.93 |
| chronic inflammatory disease* | 3 (5.7%) | 5 (8.8%) | 0.79 |
| COPD/bronchial asthma | 4 (7.5%) | 8 (14.0%) | 0.43 |
| CRT/ICD | 27 (50.9%) | 33 (57.9%) | 0.59 |
| prior valve surgery  mitral valve  tricuspidal valve  aortic valve  mitral and aortic valve | 11 (20.8%)  0 (0%)  3 (5.7%)  0 (0%) | 9 (15.8%)  2 (3.5%)  3 (5.3%)  1 (1.8%) | 0.53 |
| prior CVA | 6 (11.3%) | 9 (15.8%) | 0.69 |
| prior malign disease | 9 (17.0%) | 7 (12.3%) | 0.67 |
| history of chemotherapy/radiation | 4 (7.5%) | 4 (7.0%) | 1 |
| history of drug abuse | 1 (1.9%) | 1 (1.8%) | 1 |
| nicotine consumption  current nicotine abuse  former nicotine abuse  non-smoker  not specified | 9 (17.0%)  33 (62.3%)  5 (9.4%)  6 (11.3%) | 13 (22.8%)  23 (40.4%)  14 (24.6%)  7 (12.3%) | 0.08 |
| alcohol consumption  current alcohol abuse  former alcohol abuse  no alcohol abuse  not specified | 2 (3.8%)  4 (7.5%)  41 (77.4%)  6 (11.3%) | 4 (7.0%)  2 (3.5%)  44 (77.2%)  7 (12.3%) | 0.71 |
| infectous diseases ^§^ | 17 (32.1%) | 19 (34.5%) | 0.95 |
| intolerances ^#^ | 15 (28.3%) | 14 (24.6%) | 0.82 |

Footnote Supplementary Table 2: * includes chronical inflammatory diseases of the thoracic and abdominal organs (e.g. bronchitis, colitis) and the skin (e.g. atopic dermatitis); ^§^ from 6 weeks prior to LVAD implantation; ^#^ includes intolerances to medicines, foods and environmental factors. Categorical parameters were analysed using the Pearson χ² test or the Yates continuity correction. COPD, chronic obstructive pulmonary disease; CRT, cardiac resynchronization therapy; CVA, cerebrovascular accident; ICD, implantable cardioverter-defibrillator; LVAD, left ventricular assist device

# Supplementary Figures


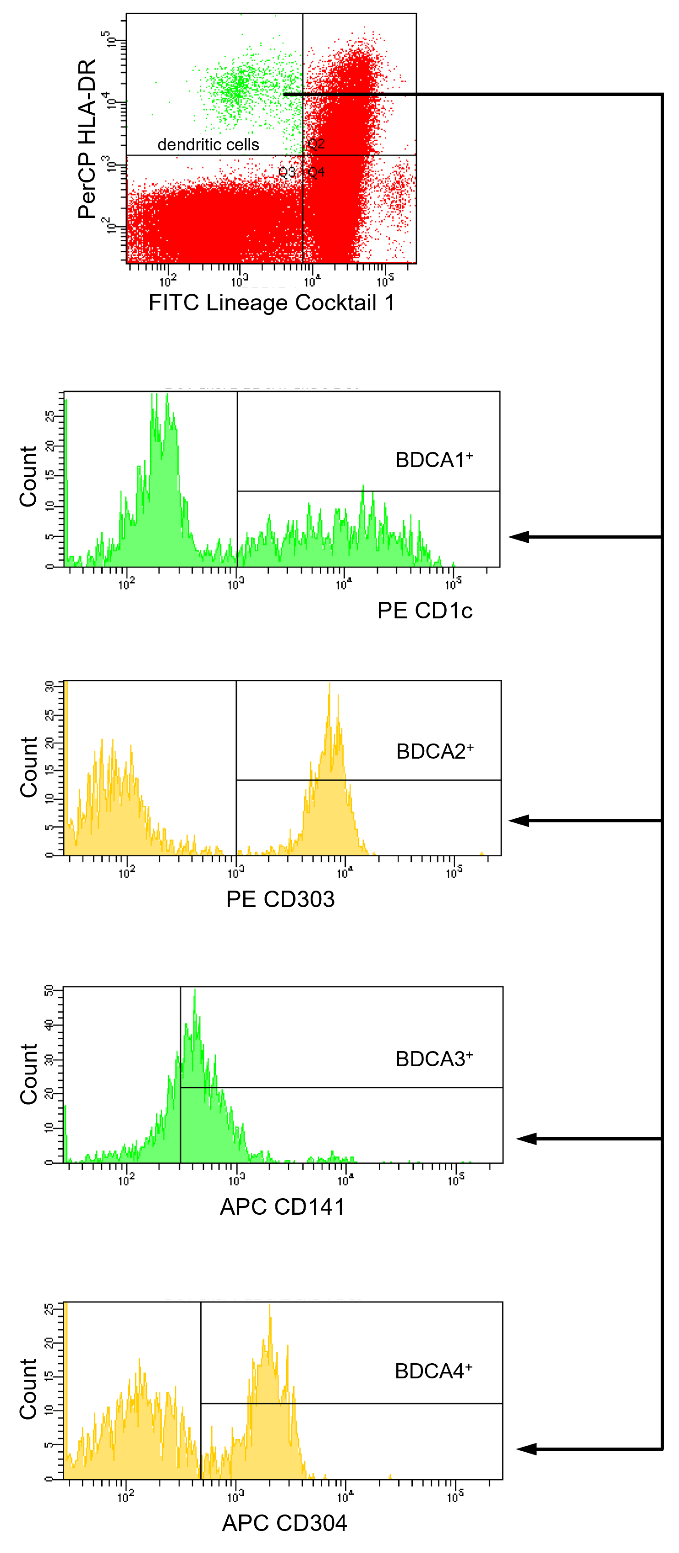


Supplementary Figure 1: Flow cytometric gating strategy for dendritic cells and their subsets positive for BDCA 1-4. Following excluding debris using a loose gate in a forward scatter-area vs. side scatter-area dot plot, HLA-DR (PerCP-labeled)-positive, lineage cocktail-1 (FITC-labeled)-negative cells were defined as dendritic cells. Staining against BDCA1 (CD1c, PE-labeled), BDCA2 (CD303, PE-labeled), BDCA3 (CD141, APC-labeled) or BDCA4 (CD304, APC-labeled) was performed to differentiate between the different subpopulations of dendritic cells. APC, allophycocyanin; BDCA1/2/3/4, blood dendritic cell antigen; CD, cluster of differentiation; DCs, dendritic cells; FITC, fluorescein isothiocyanate; HLA-DR, human leukocyte antigen DR; PE, phycoerythrine; PerCP, peridinin-chlorophyll-protein complex


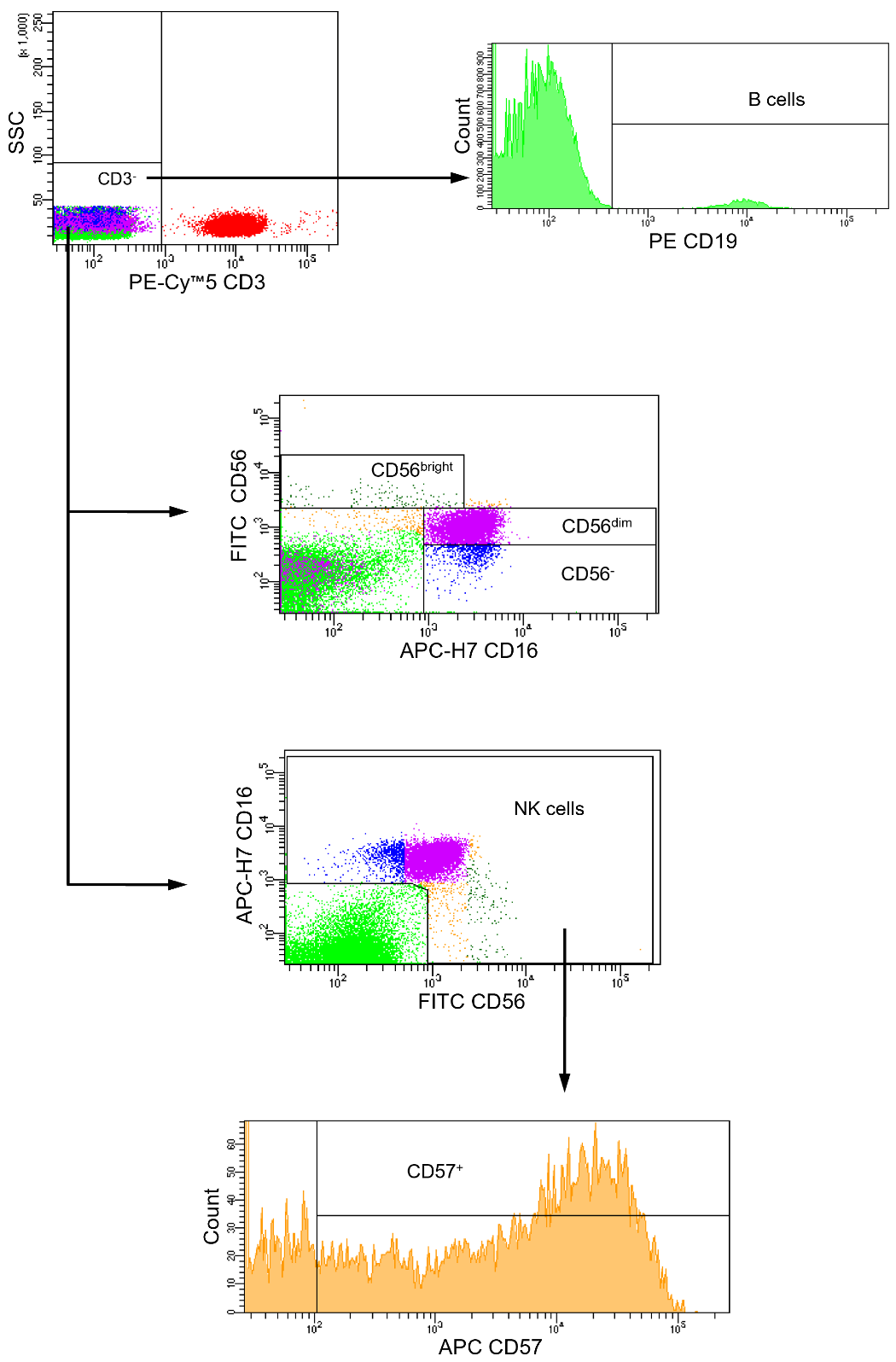


Supplementary Figure 2: Flow cytometric gating strategy for B cells, natural killer cells and their subsets. Following excluding debris using a loose gate in a forward scatter-area vs. side scatter-area dot plot and staining for CD3 (PE-Cy5-labeled), CD3^-^ cells were gated. CD19 staining (PE-labeled) of this cell population was used to identify B cells. Staining against CD56 (FITC-labeled) and CD16 (APC-H7-labeled) was performed to identify the NK cell population and their subsets (CD16^+^ CD56^-^, CD16^+^ CD56^dim^ and CD16^-/dim^ CD56^bright^). Terminal differentiation was assessed by staining NK cells against CD57 (APC-labeled). APC, allophycocyanin; APC-H7, allophycocyanin hilite 7; CD, cluster of differentiation; FITC, fluorescein isothiocyanate; NK cells, natural killer cells; PE, phycoerythrine; PE-Cy5, phycoerythrine-cyanine 5.


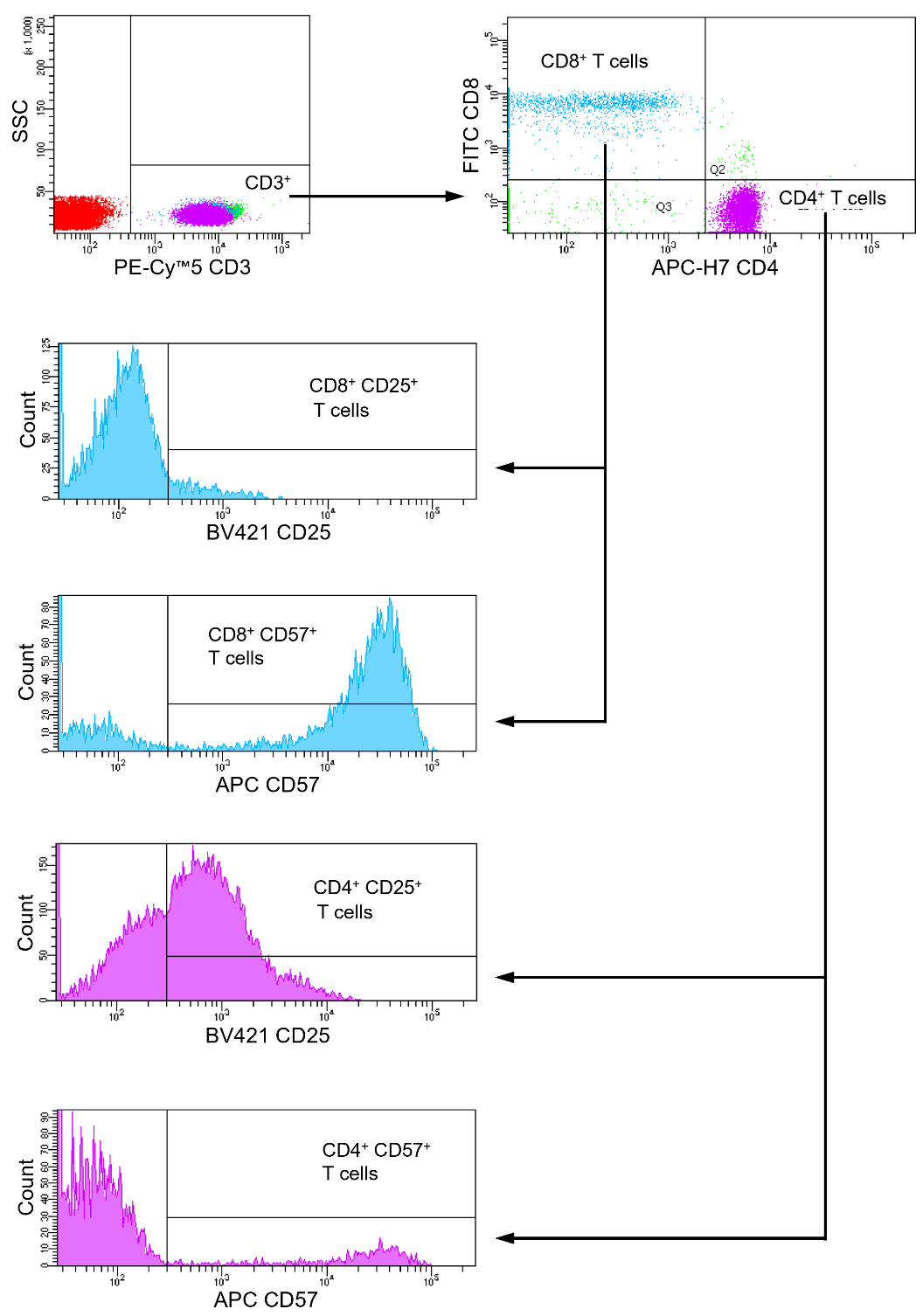


**Supplementary Figure 3: Flow cytometric gating strategy for CD4^+^ and CD8^+^ T cells and their grade of terminal differentiation and activation.** Following excluding debris using a loose gate in a forward scatter-area vs. side scatter-area dot plot, CD3 **(**PE-Cy5-labeled)-positive cells were defined as T cells. CD3^+^ T cells were subdivided into CD4^+^ and CD8^+^ cells by staining against CD4 (APC-H7-labeled) or CD8 (FITC-labeled). Terminal differentiation was assessed by staining CD4^+^ and CD8^+^ cells against CD57 (APC-labeled). Activation of CD4^+^ and CD8^+^ cells was quantified by staining CD25 (BV421-labeled). APC, allophycocyanin; APC-H7, allophycocyanin hilite 7; CD, cluster of differentiation; FITC, fluorescein isothiocyanate; PE, phycoerythrine; PE-Cy5, phycoerythrine-cyanine 5.


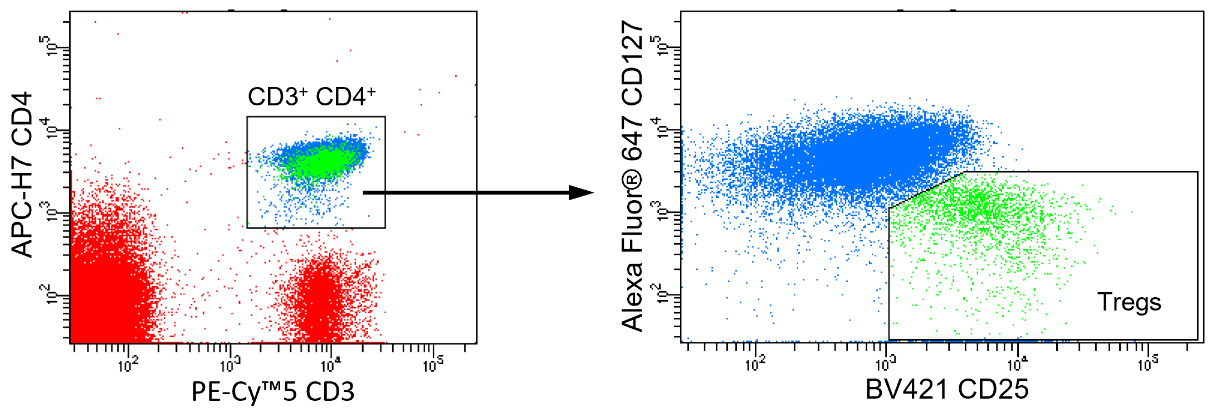


**Supplementary Figure 4:** **Flow cytometric gating strategy for regulatory T cells.** Following excluding debris using a loose gate in a forward scatter-area vs. side scatter-area dot plot, CD3^+^ **(**PerCP-Cy5-labeled) CD4^+^ (APC-H7-labeled) cells were further gated for a high CD25 (BV421-labeled) expression and a low CD127 (Alexa 647-labeled) expression. Tregs, regulatory T cells. APC-H7, allophycocyanin hilite 7; BV421, brilliant violet 421; CD, cluster of differentiation; PE-Cy5, phycoerythrine-cyanine 5.
